# Study Protocol: A Randomized Controlled Prospective Single-Center Feasibility Study of Rheopheresis for Raynaud’s Syndrome and Digital Ulcers in Systemic Sclerosis (RHEACT Study)

**DOI:** 10.3389/fmed.2022.871744

**Published:** 2022-04-14

**Authors:** Jan-Gerd Rademacher, Björn Tampe, Angela Borisch, Rosa Marie Buschfort, Andrea von Figura, Thomas Asendorf, Peter Korsten

**Affiliations:** ^1^Department of Nephrology and Rheumatology, University Medical Center Göttingen, Göttingen, Germany; ^2^Department of Medical Statistics, University Medical Center Göttingen, Göttingen, Germany

**Keywords:** rheopheresis, therapeutic plasma exchange, systemic sclerosis, Raynaud’s phenomenon, digital ulcers, blood viscosity

## Abstract

**Introduction:**

Raynaud’s phenomenon (RP) and digital ulcers (DU) are frequent manifestations of Systemic Sclerosis (SSc). Despite being very common in SSc patients, both conditions have proven to be notoriously difficult to study. There are very few available approved drugs with varying efficacy. It has been shown that the presence of DU is associated with increased whole blood viscosity (WBV). Rheopheresis (RheoP) is an extracorporeal apheresis technique used to treat microcirculatory disorders by improving blood viscosity. Improved blood flow and wound healing after RheoP treatments have been reported in single case reports.

**Methods and Analysis:**

We report the clinical trial protocol of “A *randomized controlled prospective single-center feasibility study of Rheopheresis for Raynaud’s syndrome and Digital Ulcers in Systemic Sclerosis (RHEACT).”* RHEACT aims to investigate the efficacy of RheoP on the Raynaud Condition Score (RCS) as the primary efficacy outcome measure after 16 weeks from baseline. Thirty patients will be randomized in a 1:1:1 ratio to one of two RheoP treatment groups or assigned to the standard of care (SoC) control group (intravenous iloprost). Secondary endpoints include changes in DU, changes in nailfold video capillaroscopy and patient-reported-outcomes (Scleroderma Health Assessment Questionnaire, FACIT-Fatigue, and the Disability of Arm, Shoulder, and Hand, quick version).

**Discussion:**

Apheresis techniques have been investigated in SSc but mainly in observational, retrospective studies, or single case reports. RheoP is a pathophysiologically driven potential new therapy for heavily burdened patients with SSc-associated secondary RP with or without DU.

**Ethics and Dissemination:**

The study was registered at clinicaltrials.gov (Identifier: NCT05204784). Furthermore, the study is made publicly available on the website of the German network of Systemic Sclerosis “Deutsches Netzwerk Systemische Sklerodermie (DNSS).”

## Introduction

Systemic Sclerosis (SSc) is an autoimmune disease of unknown etiology characterized by organ fibrosis and vasculopathy (1). The latter manifests clinically as Raynaud’s phenomenon (RP), present in 90–100% of patients with SSc (2). While primary RP, in which the cause is unknown, is usually benign, secondary RP occurs in the context of distinct disorders, and one should suspect a predisposing disease (3). In its classic form, Raynaud’s phenomenon leads to pallor, cyanosis, and reactive hyperemia of affected fingers and toes. A critical complication of RP is the development of digital ulcers (DU), and skin necrosis, digital (auto-)amputation, and functional impairment may occur subsequently (4). Current standard of care (SoC) treatment options aim at vasodilation. They include conservative procedures such as cold exposure prevention and hand-warming but also medical therapy with antihypertensive drugs, such as calcium-channel blockers (CCB) or the intravenous application of the vasodilating agent iloprost (1, 2, 4). However, these antihypertensive or vasodilating drugs are often not well tolerated by patients with reported side effects, including hypotension, migraine-type headache, or chest pain in up to 92% of patients (5). Further, intravenous iloprost infusions are typically performed in hospitalized patients for five to seven consecutive days and may require repeat administrations.

Other treatments, such as phosphodiesterase 5 (PDE5) inhibitors or endothelin receptor antagonists (ERA), have been studied (6) but have not been approved either for RP or DU in many countries. Bosentan, an ERA family member, has been approved for the prevention of new DU (7) but not for RP. Therefore, there is a clear need for additional treatment options.

Recently, whole blood viscosity (WBV) has been shown to be increased in a pilot study of SSc patients with DU compared to patients with a history of DU or without DU (8). Therefore, treatments that can positively affect blood viscosity might be a potential therapeutic option for patients with SSc-associated RP or DU. Plasma exchange (PEX) or variants thereof have been used in SSc with mixed results (9). In this regard, rheopheresis (RheoP) is an extracorporeal therapeutic intervention and a variant of PEX without needing a replacement fluid (fresh frozen plasma or albumin) using an additional rheofilter. It is, therefore, also referred to as double-filtration plasmapheresis (DFPP). In addition, RheoP has been investigated in other conditions affecting the microvascular circulation, such as age-related macular degeneration, sensorineural hearing loss, critical limb ischemia, or diabetic foot syndrome after failure of standard treatments (10–13).

This feasibility study aims to explore therapeutic RheoP as a novel treatment option for SSc-associated RP and DU and compare it to SoC treatment (iloprost). However, RheoP has thus far only been used in single case reports or case series (14, 15). Therefore, the optimal treatment modality, duration, or frequency of RheoP in SSc has not been established yet.

## Methods and Analysis

### Study Design

RHEACT (ClinicalTrials.gov Identifier: NCT05204784) is a randomized controlled, prospective single-center study conducted at the Department of Nephrology and Rheumatology of the University Medical Center (UMG) in Göttingen, Germany. RHEACT will compare two different RheoP treatment regimens with iloprost over 24 weeks. The primary endpoint will be assessed at 16 weeks. The decision to evaluate the primary endpoint at 16 weeks was chosen to maximize patient retention in the trial until the primary endpoint assessment. A total of 30 patients will be allocated at random to one of three treatments.

### Randomization

Patients will be randomized using block randomization with random block length, stratified for the month of inclusion (October to March or April to August) to minimize bias due to ambient temperatures on the primary outcome measure. Randomization will be performed electronically after the assessment of eligibility.

### Patients

All patients ≥ 18 years of age fulfilling the ACR/EULAR Classification Criteria for SSc (Supplementary File 1) (16) are eligible. The presence of RP with or without DU is required. Furthermore, the failure of at least one SoC therapy has to be reported. The Raynaud Condition Score (RCS), a patient-reported outcome measure used in many studies assessing RP, has to have a value ≥ 4 (17–19). To perform the RheoP procedure, appropriate venous access, either through a peripherally or centrally inserted catheter, must be established.

Exclusion criteria include significant anemia (hemoglobin < 8 g/dL), clinically relevant hemorrhagic diathesis or coagulopathy, diabetes mellitus, and severe acute or chronic kidney (eGFR < 30 ml/min/1.73 m^2^) or liver failure. In addition, patients with hypotension (systolic blood pressure < 100 mmHg) are not considered eligible. Chronic viral infections like HIV and Hepatitis B and C also preclude participation in this study. Patients with relevant neurological diseases like epilepsy, psychosis, dementia, or other relevant neurologic conditions are also excluded from participation. Other general exclusion criteria include a life expectancy of fewer than 12 months, abuse of alcohol, drugs, and a reported long-term serious tobacco abuse with documented consequential damage like severe vascular disease (Fontaine stage III or higher). Furthermore, patients with severe hyperlipoproteinemia, defined as a significant elevation of LP(a) or LDL cholesterol despite standard doses of medical therapy, are also not eligible for participation in this study. The main inclusion and exclusion criteria are summarized in Table 1.

**TABLE 1:** Inclusion and exclusion criteria.

Inclusion criteria	Exclusion criteria
>18 years of age Diagnosis of SSc according to the 2013 ACR-EULAR classification criteria of Systemic Sclerosis Presence of RP ± DU RCS ≥ 4 Failure of CCB and/or iloprost	Anemia (hemoglobin < 8 g/dL) Hemorrhagic diathesis or coagulopathy Diabetes mellitus Acute renal failure or chronic kidney damage (eGFR < 30 ml/min/1.73 m^2^) Liver failure Chronic viral infections (e.g., HIV and Hepatitis B and C) Neurological diseases like epilepsy, psychosis, dementia Life expectancy < 12 months Alcohol or drug abuse Long term serious tobacco abuse with documented severe vascular disease (e.g., Stadium Fontaine ≥ III) Hyperlipoprotemia (significant elevation of LDL cholesterol despite medical therapy)

*ACR, American College of Rheumatology; CCB, calcium channel blocker; DU, digital ulcer; eGFR, estimated glomerular filtration rate; EULAR, European Alliance of Associations for Rheumatology; HIV, human immunodeficiency virus; LDL, low-density lipoprotein; RCS, Raynaud Condition Score; RP, Raynaud’s phenomenon; SSc, systemic sclerosis.*

### Rheopheresis Procedure

**FIGURE 1:**
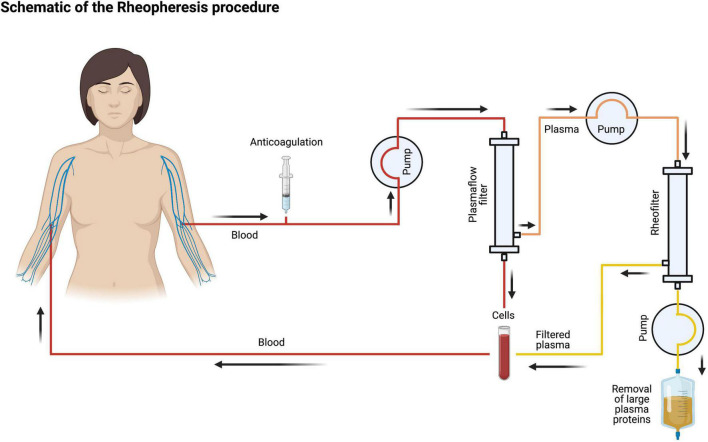
Schematic of the rheopheresis procedure. After obtaining venous access, anticoagulated blood is pumped through a plasma filter. The plasma is then run through the rheofilter, and large plasma proteins are removed. Finally, cells are reinfused, and blood is returned to the patient. The figure was created with biorender.com.

The RheoP procedure will be performed and supervised by experienced technical and nursing staff using a *Plasauto Sigma* blood purification machine (DIAMED Medizintechnik GmbH, Cologne, Germany, and Asahi Kasei Medical Co., Ltd., Tokyo, Japan). The RheoP circuit is depicted in Figure 1. When patients are cannulated peripherally, a blood flow of ∼70–80 ml/min will be used. A maximum blood flow of 100 ml/min will be used in centrally cannulated patients. The plasma flow is aimed at around 25% of the blood flow (∼25 ml/min). The target treatment volume is calculated using the formula: *42 ml × kg body weight (Example: 42 mL × 70 kg BW* = *2,940 mL [*∼*3,000 mL])*. A treatment is considered technically appropriate if a target volume of 0.8–1.0 is reached. Heparin is used as an anticoagulant to prevent blood clotting during the procedure. Typical doses are 2500 IU given as a bolus at treatment initiation and 2000 IU per hour as continuous infusion given through the apheresis machine. All products used in this study are CE certified as per regulatory requirements.

### Treatment

**FIGURE 2:**
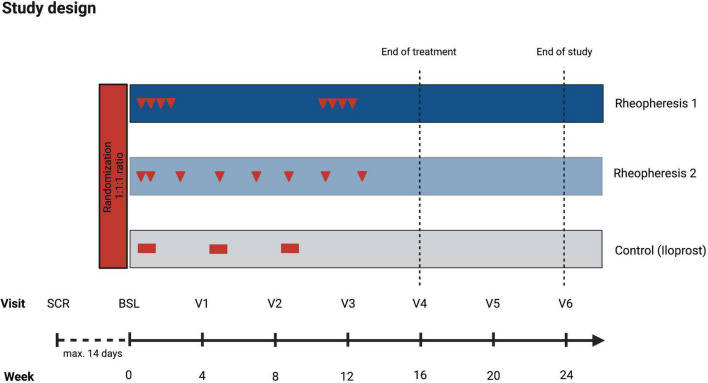
Study design. Treatment schedule (red arrows or blocks) and intervention and control groups assessments. The figure was created with biorender.com. BSL, baseline; SCR, screening; V, visit.

Patients will receive one of three treatments in a 1:1:1 ratio. The treatment groups (RheoP) will be randomized to two treatment schedules (Figure 2).

Treatment group 1: This group initially receives two rheopheresis treatments per week for 2 weeks, followed by 8 weeks without treatment. After 8 weeks, the patients will receive another 2 weeks of two treatments per week. Patients in this group will receive a total of eight RheoP treatments.

Treatment group 2: Patients in this group will receive two rheopheresis treatments in week one, followed by treatment intervals of one treatment every 2 weeks. In total, this group also receives eight treatments.

Control group: The control group is supplied with standard medical therapy for RP, consisting of intravenous iloprost therapy given as continuous infusion via an infusion pump over a minimum of 6 h (dose range 10–40 μg per day).

All patients will be advised to comply with general recommendations to avoid RP attacks, such as smoking cessation, avoidance of cold temperatures, stress reduction, and optimized skin care.

### Visit Schedule and Assessments

Study visits are performed on eight occasions: An initial screening visit to assess eligibility and a baseline visit for randomization. Then, study visits are conducted every 4 weeks up to week 24 (Figure 2). Each study visit consists of a physical examination with vital signs and evaluation of the RCS and modified Rodnan skin score (mRSS). The laboratory analyses include a complete blood count, fibrinogen, antithrombin, d-dimers, uric acid, blood urea nitrogen, creatinine, creatine kinase, nt-pro brain natriuretic peptide, troponin-I, erythrocyte sedimentation rate, low-density lipoprotein (LDL) cholesterol, immunoglobulins, protein electrophoresis, C-reactive protein (CRP), complement factor C3 and C4, and SSc-associated antibodies. All these values are either part of the routine assessment or required to evaluate the technical adequacy of the RheoP procedure (fibrinogen, albumin, IgG, IgM, LDL cholesterol).

The baseline visit and end of treatment visits include a nailfold video capillaroscopy (NVC). Other non-invasive assessments include a transthoracic echocardiography, pulmonary function testing (PFT), and a pulse wave analysis (PWA). An overview of the scheduled assessments is given in Table 2.

**TABLE 2:** Visit schedule and assessments.

Parameter	Screening visit	Baseline	Before every RheoP	After every RheoP	V16 weeks	V20 weeks	V24 weeks
**Tests and assessments**							
ICF	X						
Inclusion/exclusion criteria	X	X					
Randomization		X					
Demographic data	X						
Medical history	X						
Classification criteria ACR/EULAR 2013 for Systemic Sclerosis	X	X					
Physical examination including weight and height	X	X	X	X	X	X	X
Vital signs	X	X	X	X	X	X	X
mRSS	X	X	X	X	X	X	X
**Efficacy**							
RCS	X	X	X	X	X	X	X
PGA-VAS	X	X	X	X	X	X	X
PaGA-VAS	X	X	X	X	X	X	X
**Laboratory assessments**							
CBC	X	X	X	X	X	X	X
Immunoglobulins	X	X	X	X	X	X	X
Fibrinogen, antithrombin, d-dimers		X	X	X	X	X	X
LDL cholesterol		X	X	X	X	X	X
Protein electrophoresis		X	X	X	X	X	X
Hepatitis B,C; HIV serology	X						
SSc-associated antibodies		X	X	X	X	X	X
Complement factors C3c, C4	X	X	X	X	X	X	X
**Non-invasive assessments**							
NVC		X			X		X
PFTs		X					X
Echocardiography		X					X
PWA		X			X		X
**Patient-reported outcomes**							
SHAQ	X	X			X		X
Quick DASH	X	X			X		X
FACIT Fatigue	X	X			X		X
**Safety**							
Concomitant medication/procedures		X		Monitored after IC throughout the study until EoS			
SAEs				Monitored after IC throughout the study until EoS			

*ACR, American College of Rheumatology; CBC, complete blood count; DASH, Disability of the Arm, Shoulder and Hand; EoS; end of study; EULAR, European Alliance of Associations for Rheumatology; FACIT, The Functional Assessment of Chronic Illness Therapy; IC, informed consent; ICF, informed consent form; mRSS, modified Rodnan skin score; NVC, nailfold video capillaroscopy; PFT, pulmonary function tests; PaGA, Patient Global Assessment; PGA, Physician Global Assessment; PWA, pulse wave analysis, SAE, serious adverse events; SHAQ, Scleroderma Health Assessment Questionnaire; VAS, visual analog scale.*

### Outcomes

#### Primary Outcome

The study’s primary outcome measure is the change in RCS after 16 weeks (Supplementary File 2). The RCS is assessed at baseline and every 4 weeks before and after each RheoP (Table 2); it will also be evaluated in the control group receiving SoC therapy. The RCS incorporates the frequency, duration, severity, and impact of RP attacks on a 0–10 numerical rating scale and can be documented using paper or electronic diaries.

#### Secondary Outcomes

Secondary endpoints are the frequency of new DU, worsening of DU, time to healing of existing DU, changes of laboratory parameters, the proportion of patients with an improvement in non-invasive assessments, and changes in patient report outcome measures.

#### Patient-Reported Outcomes Measures

The assessed patient-reported outcome (PROs) measures include the patient global assessment-visual analog scale (PaGA-VAS), the German versions of the Functional Assessment of Chronic Illness Therapy (FACIT) – Fatigue Scale (Supplementary File 3), the Scleroderma Health Assessment Questionnaire (SHAQ, Supplementary File 4), and the Quick Disabilities of the Arm, Shoulder, and Hand (Quick DASH, Supplementary File 5).

#### Safety

Adverse events will be explicitly assessed at every study visit and throughout the entire after the inclusion of every subject. In addition, adverse events will be reported according to the Common Terminology Criteria for Adverse Events (CTCAE, v5.0, November 2017).

### Data Collection and Management

Clinical data for all patients, including frequency and duration of Raynaud attacks and the RCS, is collected during the routine clinic visits at least every 6 months. Study-specific data will be collected at screening, baseline, and the defined study visits (Figure 2). Data will be collected through electronic case report forms (eCRF) and stored in a provided GCP-compliant database (REDCap^®^). Data is collected in compliance with Good Clinical Practice (GCP) and following standard operating procedures (SOP) of the Clinical Trials Unit UMG to ensure high data quality.

### Statistical Aspects

#### Methods Against Bias

Selection bias is minimized by random allocation in a 1:1:1 ratio stratified by the season of admission. Block randomization with random block length will be performed. Performance and detection bias is reduced as the patient’s treatment group assignment will be concealed to a blinded team of study site investigators. Assessments will be performed before and after the treatments. In order to minimize bias related to outside temperatures, we will record the ambient temperatures during the study periods.

#### Proposed Sample Size/Power Calculations

The objective of this study is to gather initial data on the efficacy of different treatment protocols. When the sample size is 10, a two-sided 95% confidence interval for the difference in paired means of RCS will extend 1.178 from the observed mean, assuming that the standard deviation is known to be 1.9 and the confidence interval is based on the large sample z statistic. A standard deviation of 1.9 of the difference of the mean RCS was observed in prior studies on iloprost, e.g., Wigley et al. (5). Sample size calculation was performed using nQuery Version 8.3.1.0.

#### Data Analysis

Although the study has a confirmatory design that intends to test for group differences, the primary aim is to gather initial data on the efficacy of RheoP as a novel treatment option. Therefore, both treatment groups’ Pre-Post treatment effects (baseline vs. 16 weeks) will be reported with 95%-confidence intervals. Further, RCS at the end of treatment (at 16 weeks) will be compared between groups by ANCOVA with treatment group as factor and baseline RCS and season as covariates. The secondary endpoint new DUs will be compared using Poisson regression or, in the case of apparent overdispersion, negative binomial regression. Patient proportions will be summarized in tables and compared between groups using Chi Square-Test. Line plots are evaluated, where possible, to descriptively assess the influence of the intervention on observations. Estimators are calculated following the treatment policy with the intention-to-treat principle. Secondary endpoints are analyzed analogously to the primary endpoint. Finally, a sensitivity analysis with the per-protocol population will be performed. Additional vasoactive therapies, if present, will be considered as potentially confounding variables during the analysis.

## Discussion

RHEACT is the first controlled study to evaluate the efficacy of therapeutic RheoP in RP with or without DU in SSc. With this study, we seek to offer a potential new treatment option in patients with refractory RP or non-healing DU despite standard therapy. Raynaud’s phenomenon is almost universal in SSc. In our experience, most SSc patients can be managed with symptomatic or medical treatment alone. However, a significant proportion of patients require additional treatment, including iloprost, ERA, or PDE-5 inhibitors. This is supported by the latest EULAR recommendations (20), but none of these therapies is licensed for RP in SSc, and results from clinical trials have been mixed (21, 22). We acknowledge that the RCS is not a perfect primary outcome measure because it heavily relies on subjective impressions by the patients.

Nevertheless, it is currently the most widely accepted outcome measure in studies for RP. A recent survey among SSc experts showed that the RCS is mainly used in clinical trial settings and has several limitations (19): it may be subject to seasonal variation and recall bias. Also, an individual patient’s RP characteristics may change over time. We try to overcome the first limitation by block randomization according to the season of inclusion (see Methods section). Due to the relatively short observational period (24 weeks), changes over time secondary to vessel obliteration will likely not influence the results significantly. RCS also has the advantage of being a PRO.

In RHEACT, we try to gain insights regarding other secondary outcomes, such as the healing of existing DU or the development of new DU and additional PRO, including fatigue and daily function. Further, more objective outcome measures to study RP in SSc and other conditions are clearly required. For example, we recently investigated microvascular imaging (MVI) as a novel ultrasound-based method to quantify microvascular blood flow (23). However, our preliminary findings must be confirmed before applying them in clinical practice or a clinical trial setting.

Our first experiences with RheoP in refractory RP showed that it is a feasible and well-tolerated therapy (14), which may offer a novel, pathophysiologically based treatment in heavily burdened patients.

## Ethics Statement

The studies involving human participants were reviewed and approved by the Ethics Committee of the University Medical Center Göttingen, Göttingen, Germany (protocol number 36/7/21). The patients/participants will provide their written informed consent to participate in this study.

## Author Contributions

J-GR wrote the first draft and edited the manuscript. BT conceived the study and edited the manuscript. AB edited and reviewed the manuscript and is the study coordinator. RB assisted with the writing of the manuscript. AF edited and reviewed the manuscript and helped with the planning of the study. TA planned the statistical analysis and edited the manuscript. PK conceived the study, wrote the manuscript, created the figures, and acquired funding for the study. All authors contributed to the article and approved the submitted version.

## Conflict of Interest

The authors declare that the research was conducted in the absence of any commercial or financial relationships that could be construed as a potential conflict of interest.

## Publisher’s Note

All claims expressed in this article are solely those of the authors and do not necessarily represent those of their affiliated organizations, or those of the publisher, the editors and the reviewers. Any product that may be evaluated in this article, or claim that may be made by its manufacturer, is not guaranteed or endorsed by the publisher.
