# Supplementary material for: Study Protocol: A Randomized Controlled Prospective Single-Center Feasibility Study of Rheopheresis for Raynaud’s Syndrome and Digital Ulcers in Systemic Sclerosis (RHEACT Study)
Source: Front Med (Lausanne). 2022 Apr 14;9:871744. doi: 10.3389/fmed.2022.871744 (PMC9046781; doi:10.3389/fmed.2022.871744)

Supplementary Material

Study protocol: A randomized controlled prospective single-center feasibility study of Rheopheresis for Raynaud’s syndrome and Digital Ulcers in Systemic Sclerosis (RHEACT study)

Jan-Gerd Rademacher^1#^, Björn Tampe^1#^, Angela Borisch^1^, Rosa Marie Buschfort^1^, Andrea von Figura^1^, Thomas Asendorf^2^, Peter Korsten^1^*

^1^Department of Nephrology and Rheumatology, University Medical Center Goettingen, Germany; ^2^Department of Medical Statistics, University Medical Center Goettingen, Germany

^#^JGR and BT contributed equally

***Correspondence:**Dr. med. Peter Korsten
peter.korsten@med.uni-goettingen.de

## ACR/EULAR classification criteria for the classification of systemic sclerosis (1)


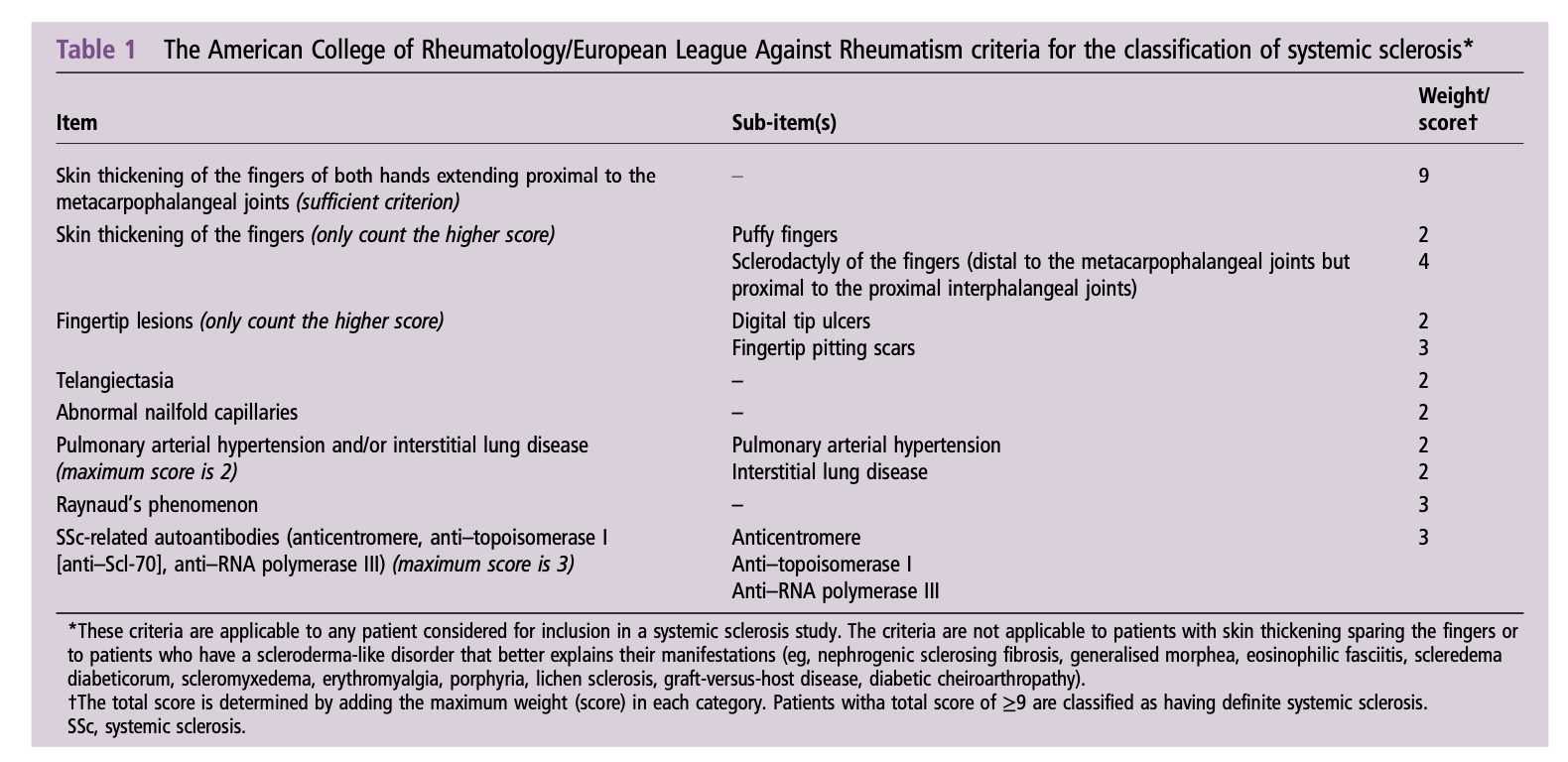


## Raynaud Condition Score (2)


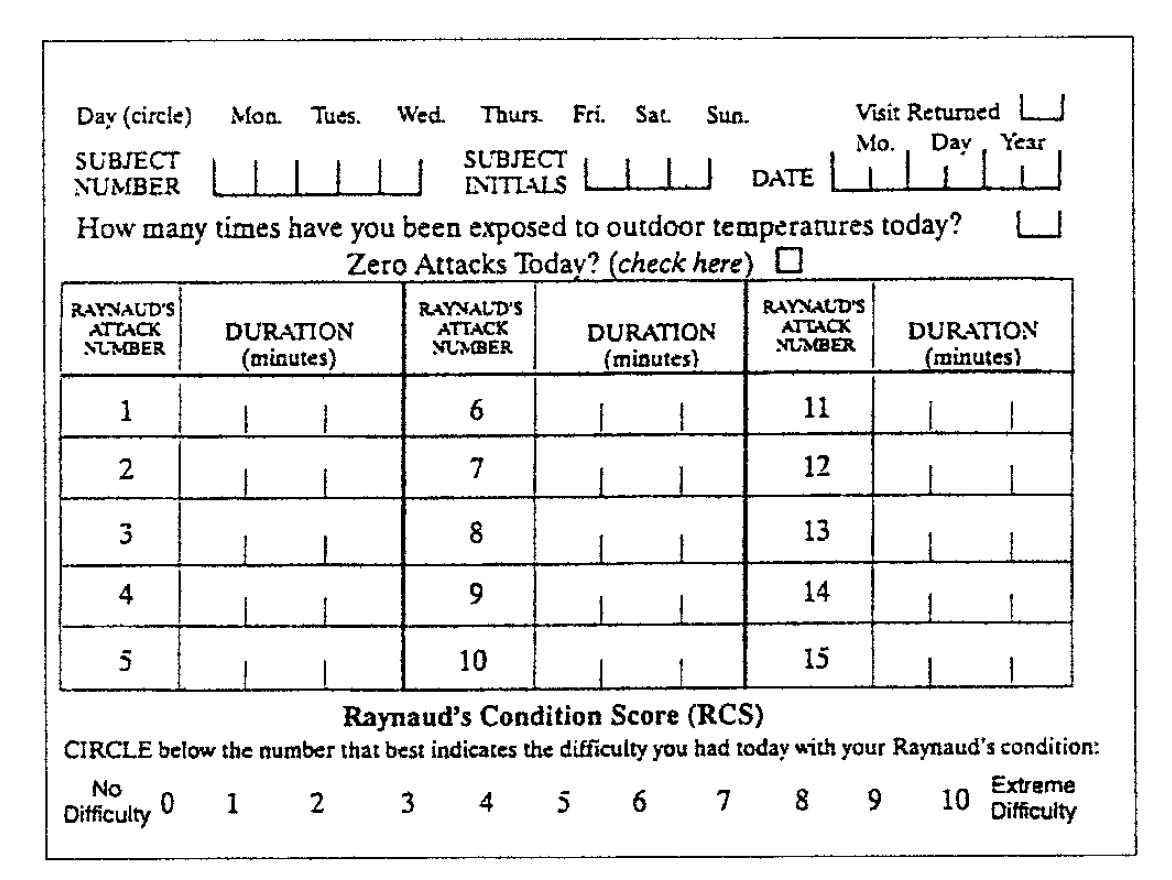


## Scleroderma Health Assessment (German version)


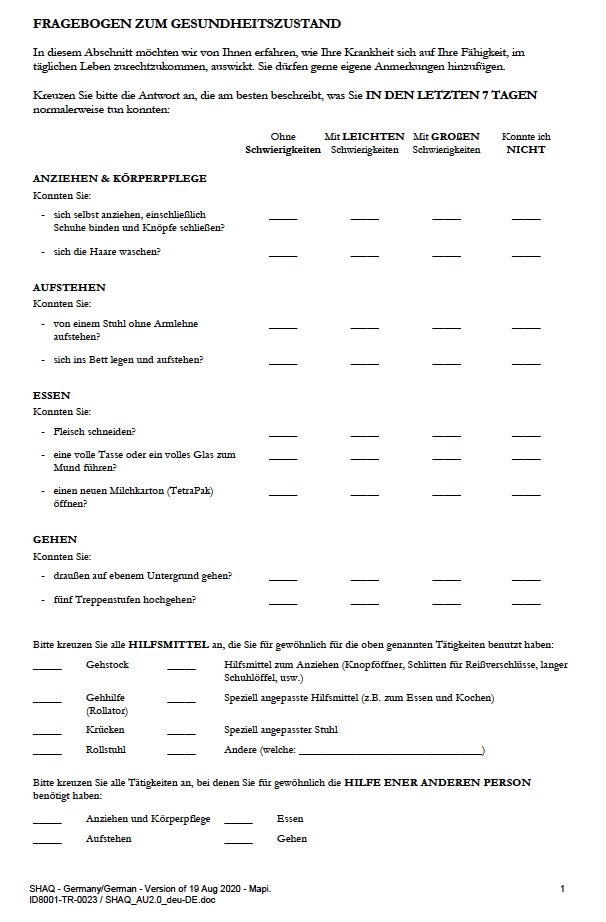


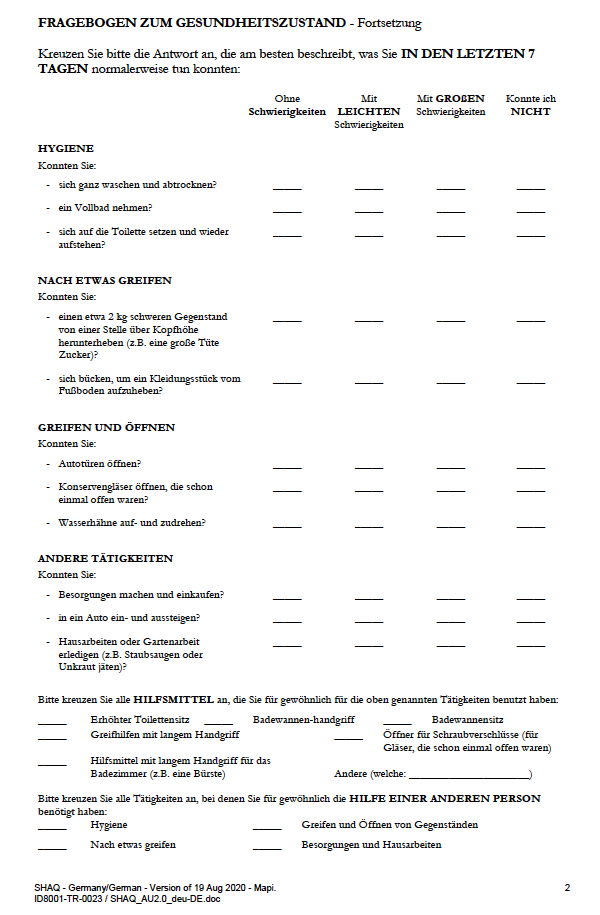


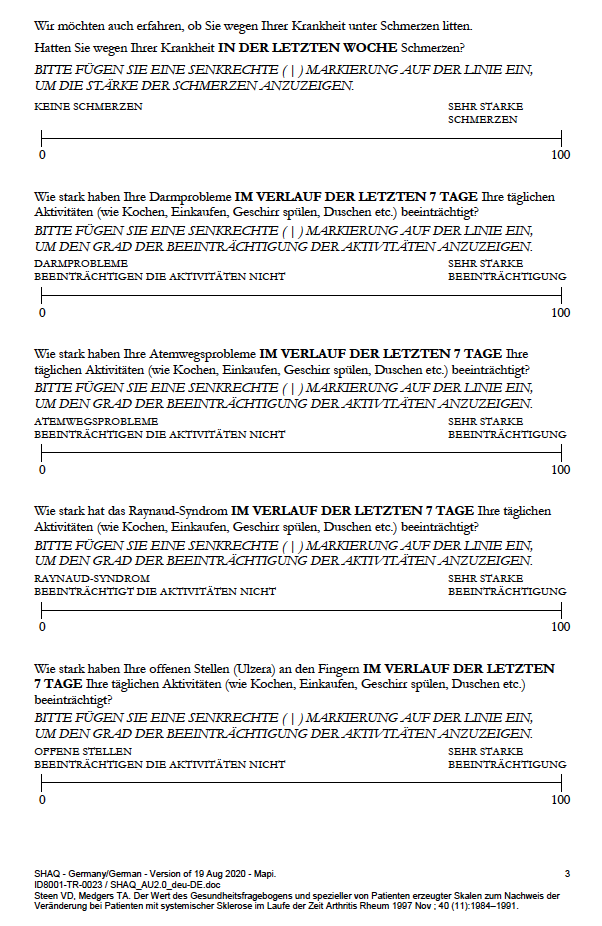


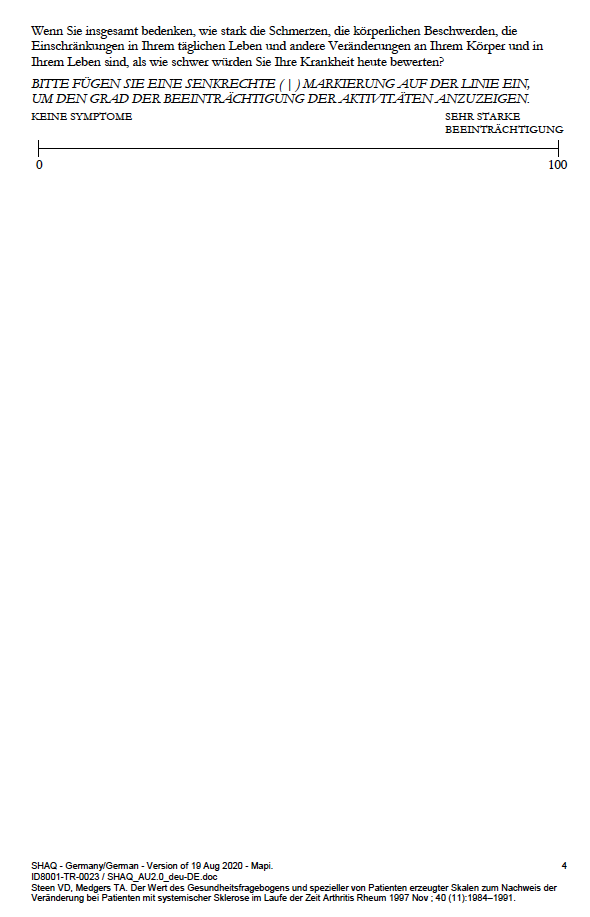


## FACIT Fatigue Score (German)


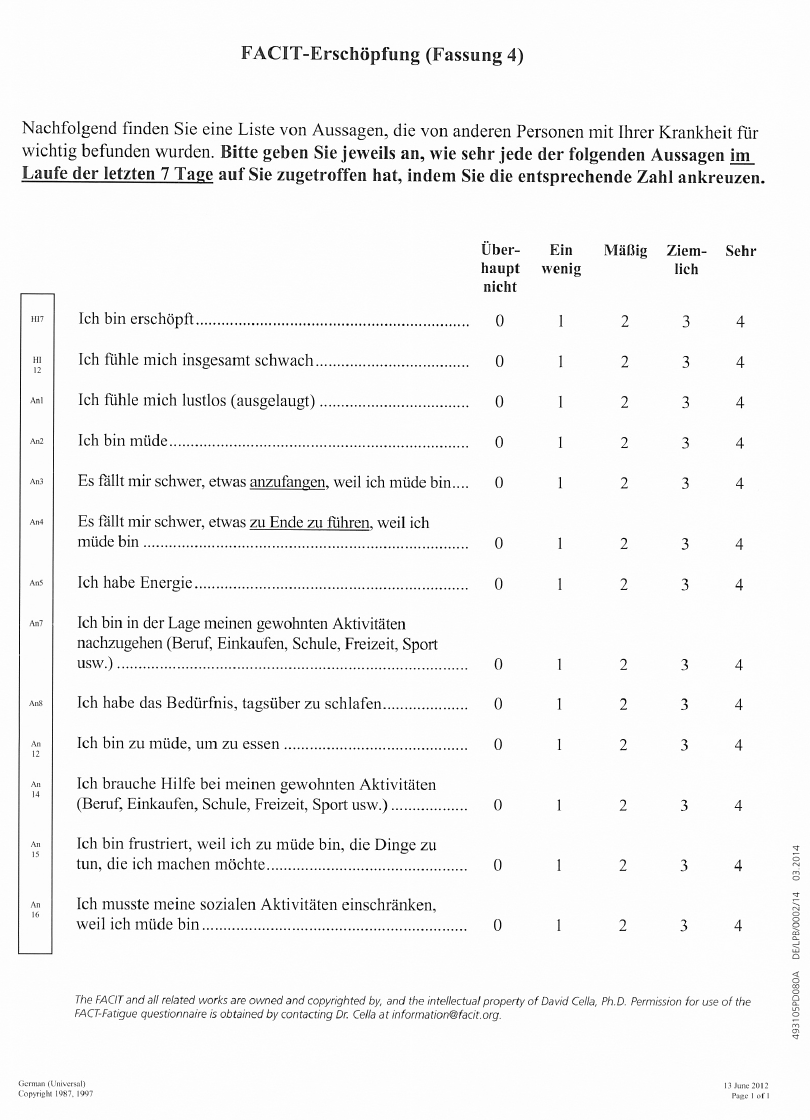


## Quick DASH (German)


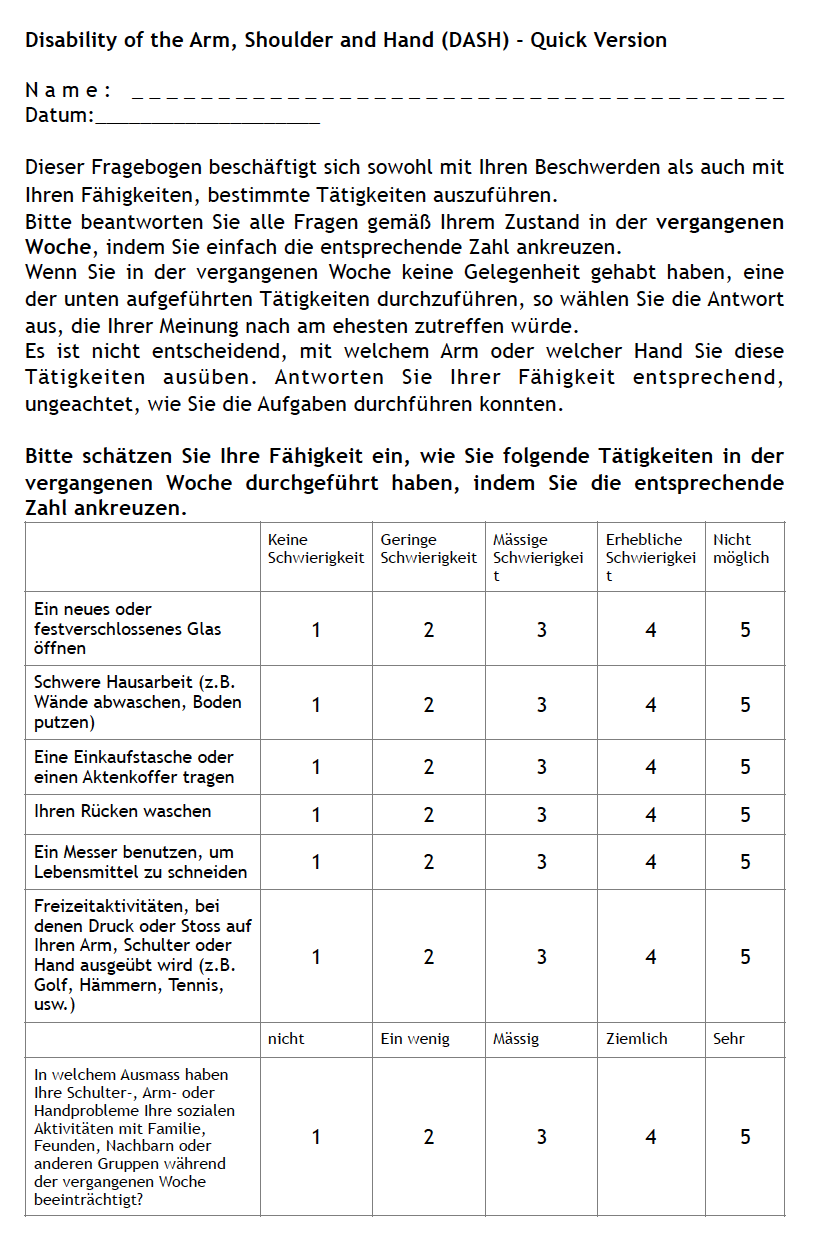


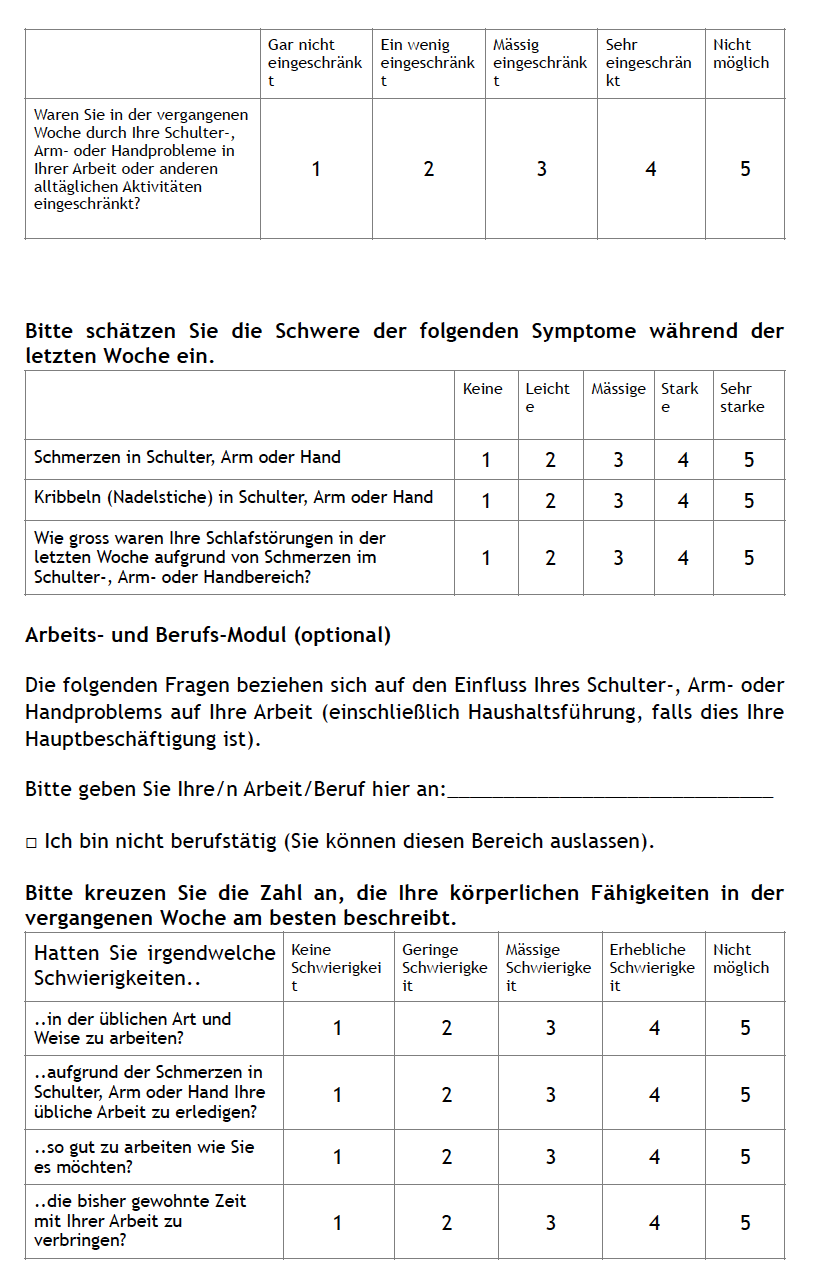

Supplement: Supplementary file 1 [file Data_Sheet_1.DOCX]
